# Cervical Microbiota Influences Cytokine Diversity in Cervical Intraepithelial Neoplasia among Rural Women in the Akyemansa District of Ghana

**DOI:** 10.1155/2023/5129709

**Published:** 2023-08-17

**Authors:** Loretta Betty Blay Mensah, Sebastian Ken-Amoah, Mainprice Akuoko Essuman, Betty Anane-Fenin, Evans Kofi Agbeno, Sebastian Eliason, Samuel Essien-Baidoo

**Affiliations:** ^1^Department of Microbiology and Immunology, School of Medical Sciences, College of Health and Allied Sciences, University of Cape Coast, Cape Coast, Ghana; ^2^Department of Obstetrics and Gynaecology, School of Medical Sciences, College of Health and Allied Sciences, University of Cape Coast, Cape Coast, Ghana; ^3^Department of Medical Laboratory Science, School of Allied Health Sciences, College of Health and Allied Sciences, University of Cape Coast, Cape Coast, Ghana; ^4^Department of Community Medicine, School of Medical Sciences, College of Health and Allied Sciences, University of Cape Coast, Cape Coast, Ghana

## Abstract

**Background:**

In recent times, cervical dysbiosis which mostly causes and aggravates infections is highlighted for its role in immune modulation in cervical dysplasia, which promotes the shifting of Th1 phenotype immunity to Th2 phenotype immunity. This study therefore estimated and compared the levels of circulatory IL-4, IL-6, IL-10, TNF-*α*, and IFN-*γ* cytokines among adult women identified to have different grades of cervical intraepithelial neoplasia (CIN) and with cervicovaginal infection.

**Methods:**

A total of 157 participants were recruited from the Akyemansa District of Ghana, and cervical swabs and blood samples were taken. The Pap smear test, microbiological culture, and ELISA were employed for cytology analysis, bacteria isolation, and identification and estimation of IL-4, IL-6, IL-10, TNF-*α*, and IFN-*γ* cytokines, respectively.

**Results:**

Overall, 14/157 (8.9%) had CIN with 7.6% having CIN 1 and 1.3% having CIN 2. The main predictor for CIN was age above 46 years (OR 11.16, 95% CI: 2.4-51.8). Bacterial vaginosis (*p* = 0.003) and Candida infection (*p* = 0.012) were significantly higher in CIN. Again, *Staphylococcus aureus* (60% vs. 17.6%, *p* = 0.005)*, Citrobacter* sp. (40.0% vs. 13.2%, *p* = 0.017), and *Morganella morganii* (40.0% vs. 4.4%, *p* = 0.002) isolates were significantly higher in CIN-positive participants. IL-10 and TNF-*α* concentrations were elevated in participants with CIN 1+ (TNF-*α* NIL vs. CIN 1+ only, *p* < 0.05) while IL-6 was decreased among participants with CIN 1+. In the presence of vaginal infection, TNF-*α* decreased among CIN 1+ participants while IL-10 remained elevated.

**Conclusion:**

The findings of this study suggest that cervical dysbiosis causes immune suppression, which creates a suitable microenvironment for the development of CIN.

## 1. Introduction

Over the past decade, the global burden of cervical cancer has gradually been declining; however, the burden in low- and middle-income countries such as Ghana continues to be of great public health concern [1, 2]. Currently, it ranks as the fourth most common cancer diagnosed among women [3, 4] of which the incidence in low- to middle-income countries, such as Ghana, Brazil, and India, contribute to over 80% of the global burden [4, 5].

Previous studies have established the role of persistent HPV infection in the development of precancerous lesions and invasive cancer [1, 6–9]. To achieve this progression, immunological evidence points to an alteration of cell-mediated immunity in which there is a shift of Th1 phenotype cytokine response to Th2 phenotype cytokine response [10, 11]. Thus, it has been observed that there is a reduction in cytokines such as interferon- (IFN-) *γ* and tumour necrotic factor (TNF-*α*) and an increase in interleukin- (IL-) 10 and IL-4 [10, 12]. Similarly, IL-6 is increased in CIN, especially CIN I [12]. As anti-inflammatory cytokines, IFN-*γ* and TNF-*α* are immunostimulatory and have antitumour properties [12, 13]. IL-6 cytokine in CIN, then, acts as inflammatory via the transsignalling pathway to be antiapoptotic and also increases the expression of vascular endothelial growth factor (VEGF) via the STAT3 pathway to promote cancerogenesis [14–16]. IL-4 and IL-10, on the other hand, are immunoinhibitory cytokines which promote tumour growth [10, 12].

In recent times, vaginal dysbiosis is being studied for its role in the possible modulation of immune response in CIN [17–21]. Vaginal dysbiosis is closely related to gynecological infections as the microflora imbalance contributes to the overgrowth of anaerobic bacteria mostly implicated in bacterial vaginosis (BV), aerobic bacteria also implicated in aerobic vaginosis (AV), and occasionally cytolytic vaginosis [22, 23]. Studies have demonstrated the involvement of specific bacteria genera, such as *Fusobacterium* and *Pseudomonas*, in immune suppression in CIN [17, 24]. Also, sialidase secreted by *Gardnerella* is reported to promote the progression of cervical lesions in persistent HPV infection and precursor lesions [25, 26].

Currently, there remains a paucity of data on the prevalence and immune response of cervical cancer in Ghana. Again, the role of cervicovaginal microbiota in the occurrence and progression of CIN is not well understood. This study therefore (1) identified the proportion of the rural population with different grades of cervical intraepithelial lesions (CIN), (2) isolated and identified cervicovaginal pathogens within the microenvironment of the participants, and (3) estimated and compared the concentrations of IL-4, IL-6, IL-10, TNF-*α*, and INF-*γ* of participants with CIN to the normal population.

## 2. Methods and Materials

### 2.1. Study Design and Subjects

This was a cross-sectional study that was conducted in five (5) rural communities in the Akyemansa District of the Eastern Region of Ghana. A total of 157 participants who consented to their biological samples being taken were recruited for the study. Data collection was done in May-June 2019.

### 2.2. Study Site and Population

The Akyemansa District of the Eastern Region of Ghana is a predominantly rural setting with farming and mining activities being the major occupation. According to a recent population and housing census, the district's estimated female population was 61,993, representing 50.5% of the district's overall population [27]. In respect to climate conditions, the region has an average temperature of 27°C, with an average annual rainfall of 1,450 mm between 85 and 130 days [28]. Members of the district rely on pit latrines, boreholes as the main sources of toilet and water facilities, and open-space public dump for waste disposal. We have recently reported poor knowledge of human papillomavirus and cervical cancer in the area [29, 30].

### 2.3. Sample Size and Sampling

The sample size was calculated using the Cochran formula with a prevalence of 7.51% based on data reported by Obiri-Yeboah et al. [31], a confidence level of 95%, and a precision of 5%. A calculated sample size of 107 was obtained. A house-to-house visit was employed, and the women were educated on cervical cancer and the need for regular screening. Using a simple random sampling strategy, the second (2^nd^) adult female in every second household was recruited, and where the 2^nd^ adult female declined to participate in the study, the immediate next available person is recruited upon her consent. Thus, a total of 274 participants were recruited. Specific dates were communicated to the participants for a visit to their community health centers for their biological samples to be taken. On the said dates, only 157 participants consented for their biological samples to be taken and were recruited for the study.

### 2.4. Data and Sample Collection

A structured pretested questionnaire [31, 32] was used to collect sociodemographic and socioeconomic data and the risk behaviours of participants. Cervical smears for the Papanicolaou test [33] and two high vaginal swabs (A and B) for bacteriology were taken by three trained female midwives assigned to the study, using Ayre's spatula and cotton swab sticks, respectively. Additionally, 3 ml of venous blood was taken from the participants into serum separation tubes (SST) for cytokine estimation. The blood samples were taken in a well-lit area by locating any visible vein (median cubital vein or cephalic vein or basilic vein) on the arm of the participants. With gloved hands, a tourniquet was then tied at 4 finger widths above the venipuncture site, and the entry site was disinfected with a 70% alcohol swab. Carefully, a sterile needle was inserted into the vein after the alcohol had dried, and 3 ml of blood was drawn into the syringe. The tourniquet was released, and the needle was gently drawn out of the vein while a dry cotton was pressed on the entry site to initiate clotting. The blood was dispensed into the SST by inserting the needle into the tube's stopper, and without pressure, the blood was slowly dispensed into the tube. The syringe was discarded into puncture-resistant sharp boxes, and the tubes were labelled with the participant's generated pathological number. Finally, a phlebotomy plaster was placed on the entry site of the venipuncture, and the participant was allowed to leave [34].

The cervical smear was fixed with alcohol, whereas the two vaginal swabs were kept on ice and transported to the laboratory, with one swab (sample B) each kept in a transport medium (modified buffered peptone water: pH 7.2). In the laboratory, swab A was immediately prepared for wet prep and Gram staining. The wet prep was observed for pus cells and the presence of trichomonads, yeast-like cells, and clue cells. Vaginal infection was considered for samples with 10/high-power field (HPF) and more pus cells, and it was confirmed with growth from the culture. Sample B was inoculated on blood and chocolate agar plates.

The venous blood samples in the SST were centrifuged at 4000 rpm for 5 minutes, and the serum was aliquoted into 1.5 ml Eppendorf tubes and stored at -20°C. The serum was later thawed and diluted for cytokines (IL-4, IL-6, IL-10, TNF-*α*, and IFN-*γ*) estimation using enzyme-linked immunosorbent assay (ELISA) for IL-4 (Sunlong Biotech, Cat. No.: SL0997Hu), IL-6 (Sunlong Biotech, Cat. No.: SL1001Hu), IL-10 (Sunlong Biotech, Cat. No.: SLD003Hu), TNF-*α* (Sunlong Biotech, Cat. No.: CSL1761Hu), and IFN-*γ* (Sunlong Biotech, Cat. No.: SL0960Hu).

All women found to have abnormal reports with the cytology were referred to the gynecologist for further evaluation and management at their own cost.

### 2.5. Cervical Cytology

As described previously, a standardized protocol for Papanicolaou (Pap) staining was followed [35]. Cervical smears were prepared in the laboratory and read by a cytopathologist at Korle-Bu Teaching Hospital (KBTH) using the Bethesda 2014 guidelines for CIN classification [36, 37]. The squamous epithelial cells' abnormalities were identified as low-grade squamous intraepithelial lesions (also CIN 1) with mild dysplasia and koilocytosis, and high-grade squamous intraepithelial lesions (comprising CIN 2 and CIN 3) which are marked with nuclear enlargement and hyperchromasia. The nuclear irregularities reflect profound derangements in cellular function and genetic instability [37].

### 2.6. Isolation and Identification of Organisms

Following aseptic standard isolation techniques, swab B was inoculated on blood and chocolate agar plates by gently swirling them on the agar's exterior to create pools and streaking from the pool clockwise to obtain discrete colonies of microorganisms. The blood agar plates were aerobically incubated at 35 ± 2°C for 18-24 hours while the chocolate agar plates were anaerobically incubated at 35 ± 2°C for 18-24 hours in tightly covered jars with lighted candle sticks. The plates were then observed for colony growth and identification.

For identification, colonial morphology, nature of haemolysis on the blood agar, Gram staining to identify bacteria gram type, and reactions to various biochemical tests were employed. The biochemical tests used were triple sugar iron (TSI), catalase, coagulase, urease, citrate, oxidase, and indole tests. The Gram-stained slides were also observed for the presence of leucocytes, clue cells, and “false clue cells” which are suggestive of cytolytic vaginosis.

### 2.7. Diagnosis of Bacterial Vaginosis (BV)

As described in a previous study [38], the diagnosis of BV was established using the Amsel criteria; particularly, the presence of clue cells in wet mount preparations and Gram-stained smear, the presence of thin watery homogenous vaginal discharge, and fishy odour were considered. The fishy odour was detected using the Whiff test whereby 15 drops of 10% KOH were added to vaginal discharge transferred on a microscope slide.

### 2.8. Diagnosis of Candida Infection

This was established by the presence of five or more (≥5) budded yeast-like cells/high power field (HPF) in the wet mount preparation and the Gram stain as well.

### 2.9. Estimation of IL-4, IL-6, IL-10, TNF-*α*, and IFN-*γ* Cytokines

Estimation of the cytokines in thawed diluted serum (dilution factor of 5) was done using commercial ELISA kits for IL-4 (Sunlong Biotech, Cat. No.: SL0997Hu), IL-6 (Sunlong Biotech, Cat. No.: SL1001Hu), IL-10 (Sunlong Biotech, Cat. No.: SLD003Hu), TNF-*α* (Sunlong Biotech, Cat. No.: CSL1761Hu), and IFN-*γ* (Sunlong Biotech, Cat. No.: SL0960Hu). Following the manufacturer's protocol, the outlined procedures were carefully noted, and finally, the optical density (OD) absorbances of the strip plates were read at 450 nm using a microtiter plate reader (Biobase, China) within 15 minutes after adding the Stop Solution. The concentrations of the samples were calculated with the aid of a standard curve and linear plots obtained from the known concentrations against the optical densities of the standards. The sensitivity of the IL-4, IL-6, IL-10, TNF-*α*, and IFN-*γ* ELISA kits were 0.8 pg/ml, 0.5 ng/l, 0.3 pg/ml, 5.5 pg/ml, and 8.0 pg/ml, respectively.

### 2.10. Data Analysis

Data obtained were entered into Microsoft Excel (2016) and transferred into STATA Software version 14 (STATA Corp, Texas USA) and GraphPad Prism 8 (GraphPad Software, San Diego, California, USA) for statistical analysis. A descriptive analysis was done on the sociodemographic and socioeconomic characteristics as well as other relevant characteristics. Bivariate analysis was done for cytological abnormalities. The means of cytokine levels were analyzed and compared using a two-way analysis of variance (ANOVA). All means were reported as mean ± standard deviation (SD). Statistical significance was considered when the *p* value was less than or equal to 0.05 (*p* value ≤ 0.05).

### 2.11. Ethical Consideration

Ethical approval was obtained from the Institutional Review Board of the University of Cape Coast (UCCIRB/CHAS/2019/30) before the start of the study. Additionally, a community-entry durbar was organized in each of the selected communities to seek the consent of the leaders of the communities. Informed and written consent was obtained from the participants, with their anonymity, and confidentiality maintained. Furthermore, the researchers were available to answer questions arising from the respondents, while they assisted those who could neither read nor write to complete their questionnaires.

## 3. Result

### 3.1. Participants' Characteristics and CIN Prevalence

**Figure 1:**
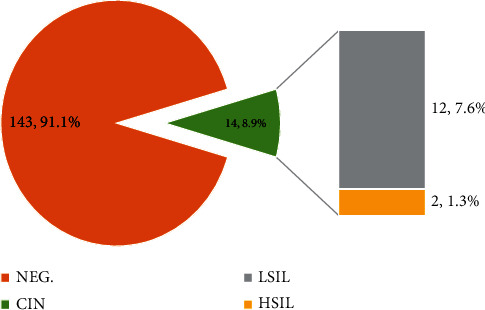
Proportion of participants with epithelial squamous cell abnormalities. Abbreviations: NEG = no squamous intraepithelial lesions seen; LSIL = low-grade squamous intraepithelial lesion (CIN 1); HSIL = high-grade squamous intraepithelial lesion (CIN 2).

Among the total of 157 participants, 14 (8.9%) of them had various grades of cervical intraepithelial neoplasia: 12 (7.6%) and 2 (1.3%) for CIN 1 and CIN 2, respectively (Figure 1). The demographic and behavioural data of participants recruited for the study are detailed in Table 1. The average age of participants was 41.2 ± 1.05. All the participants with CIN 1 and advanced cytopathology (CIN 1+) were 41 years of age and older (≥41 years old) and were also living with a life partner at the time of the study. Again, almost all the CIN 1+ participants have had the peak of their education at the basic school (JHS or primary school), were economically engaged, and earned GhȻ 500.00 (≈ USD 50.00) or less. With regard to behavioural characteristics of the participants that are associated with CIN 1+, almost all the participants had their sexual debut at 20 years of age and below (≤20 years, 93%), have had a maximum of three lifetime sexual partners (≤3, 93%), had ever taken oral hormonal contraceptives for a period of at least 3 years within the past 10 years (64%), and have had multiparity (100%).

### 3.2. Predictors of CIN among Participants

The main predictor of CIN was old age (>46 years, *p* < 0.05). However, other factors which did not show statistical significance such as having a sexual debut at 20 years and below, having ever taken an oral hormonal contraceptive for at least a period of 3 years, and having parity ≥ 4 were identified to positively predict the occurrence of CIN (Table 2).

### 3.3. Cervicovaginal Infection among Participants

Over 60% of the participants had bacterial infections, 31% had Candida infection, and 9% had bacterial vaginosis. Candida infection and bacterial vaginosis were significantly higher among participants with CIN 1 as shown in Table 3. Of the bacteria isolates, *Escherichia coli* (31.7%)*, Staphylococcus aureus* (21.8%), and *Citrobacter* sp. (15.8%) were the most common isolates found among participants. In CIN-positive participants, *Staphylococcus aureus* (60% vs. 17.6%, *p* = 0.005)*, Citrobacter* sp. (40.0% vs. 13.2%, *p* = 0.017), and *Morganella morganii* (40.0% vs. 4.4%, *p* = 0.002) isolates were significantly higher compared to CIN-negative participants. *Lactobacillus* spp. and *Pseudomonas* sp. were not isolated in CIN (Table 3).

### 3.4. Levels of Inflammatory Cytokines

As shown in Table 4, IL-10 and TNF-*α* concentrations were elevated in participants with CIN 1+ (TNF-*α* NIL vs. CIN 1 only, *p* < 0.05) while IL-6 was decreased among participants with CIN 1+. Lastly, in the presence of vaginal infection, TNF-*α* decreased among CIN 1+ participants while IL-10 remain elevated (*p* > 0.05) as shown in Table 5.

## 4. Discussion

The relationship between dysbiosis and immune modulation has become a very important direction of study especially in cervical cytopathology and other related genital inflammation that increases a woman's susceptibility to infections [20, 21, 39, 40]. In this study, we (1) identified the proportion of a rural population with different grades of cervical intraepithelial neoplasia (CIN), (2) isolated and identified cervicovaginal pathogens within the microenvironment of the participants, and (3) estimated and compared the concentrations of IL-4, IL-6, IL-10, TNF-*α*, and INF-*γ* among participants with CIN to know the trend of the immune response.

In this study, involving 157 participants with a mean age of 41.2 ± 1.05 years, 14 (8.9%) of the participants had different grades of CIN—with 2 (1.3%) being high-grade squamous intraepithelial lesions. The most significant predictor of cytopathology among the participants was older age (>46 years). Candida infection and bacterial vaginosis were the predominant infections among the participants with CIN. Finally, vaginal infection among the participants with CIN expressed immune modulation which favoured immunosuppression with an increased level of IL-10.

The prevalence of CIN in women is of particular relevance because these lesions can develop into cervical cancer. The detection of an 8.9% prevalence of CIN with 7.6% and 1.3% being CIN 1 and CIN 2, respectively, was comparable to earlier studies in the Ashanti and Central Regions of Ghana, which reported a prevalence of CIN between 3.5-12.6% and 1.2-14.1%, respectively [31, 41]. In China, CIN 1 and CIN 2 pooled prevalence of 3.4% and 1.5% has been reported [42]. Contrary to our study, Ntuli et al. [43] reported a higher proportion (19.2%) of abnormal cytology among participants in South Africa. This finding reflects differences in geographical location and lifestyle among the participants. Again, the study from South Africa involved HIV-infected women; thus, a high prevalence is expected as HIV infection may increase an individual's risk of having CIN [44]. South Africa's national HIV/AIDS prevalence of 14% is higher than that of the national prevalence of Ghana, 2% [45, 46]. The frequency of CIN in this population should influence cervical cancer awareness creation and screening practices in the community and other places in Ghana.

We found old age (>46 years old) as the main factor associated with abnormal cytology among participants (OR 11.16, 95% CI: 2.402-51.844). Aging and childbirth have been identified to cause transformational changes in the cervix [47]. Other related reasons for this finding are the insidious onset and progression of cervical dysplasia which usually spans within a 10–20-year lap towards carcinogenesis and reduced immunity in aging [48–50]. This finding was inconsistent with that of studies done in Zambia and Tanzania which identified the main predictors of abnormal cytology as the presence of hr-HPV infection and a history of having multiple sexual partners, respectively [32, 51].

Almost two-thirds (64.3%) of the participants had bacterial infections with the participants with CIN being the group with significantly higher bacterial vaginosis (BV) and Candida infection (*p* = 0.003 and *p* = 0.012, respectively). This finding was consistent with that of studies in Iran and China [52, 53]. It is reported that in cervical dysplasia and cervicovaginal infections, the latter creates a conducive microenvironment for the former and vice versa [52]. In addition, BV caused by *Gardnerella vaginalis* enhances cervicovaginal infections with the secretion of an enzyme, sialidase, which breakdown the vaginal mucus by cleaving its glycoproteins thus breaking the barrier to inhibit bacteria-host interaction [26].

With regard to the predominant bacteria isolates among participants with CIN, *Staphylococcus aureus*, *Morganella morganii*, and *Citrobacter* spp. were identified. Consistent with earlier studies, *S. aureus* and other aerobes such as *E. coli* and *Morganella* spp. have been associated with aerobic vaginitis [54]. Again, this finding was similar to that of previous studies [55, 56]. Cervical dysbiosis has the propensity to increase cervical pathology partly due to its ability to cause cervical inflammation [57].

Regarding the natural immune response towards cervical dysplasia, the distribution of the cervicovaginal microbiome plays a crucial role in its modulation [26]. In this study, immune modulation was observed with decreased TNF-*α* among participants with cervical lesions and coinfected with other reproductive tract infections with a statistical significance between participants with no infection and those with only vaginal infections (54.32 ± 9.50 vs. 31.99 ± 19.29 pg/ml, *p* < 0.00001) and between those with RTI and those with CIN 1+RTI (31.99 ± 19.29 vs. 48.73 ± 18.77 pg/ml, *p* < 0.05). We also found an increased IL-10 among the participants with lesions and vaginal infections. The increase in IL-10 demonstrates a Th2-like immune response in which there is immune suppression favouring a suitable microenvironment for lesion progression and tumorigenesis [13, 17, 58, 59]. The findings of this study were similar to that of an earlier study [17] which similarly demonstrated that cervical microbiota is a possible modifier of the cytokine profile of the cervical microenvironment during the development of cervical neoplasm.

We are aware of the limitations of our study which have to be considered in interpreting the findings of the study. First, our sample is medium-sized, which limits some of our inferences. Secondly, microbial determination depended solely on culture techniques which have the potential to miss some microbes unlike the more sensitive molecular techniques. Thirdly, the microbial examination was based on only one sample collected from participants, and thus, some isolates were likely missed. A previous report has suggested an average of 29 samples per participant to examine the association between HPV infections and the vaginal microbiome [60]. A larger cohort study incorporating molecular techniques will be needed to expand and validate our findings. In addition, mechanistic studies using in vitro and in vivo models would be required to fully understand the complex relationships between cervical microbiome diversity, immune response, and HPV infections.

## 5. Conclusion

The study shows that a significant proportion of women in the rural population have various grades of cervical intraepithelial neoplasia; thus, more robust screening programs should be organized and made accessible to Ghanaian women. We also identified that cervicovaginal infection is capable of immune-modulating immune responses by shifting Th1-type immunity to Th2-type immunity. The findings of this study suggest that cervical dysbiosis via infection causes immune suppression, which could create a suitable microenvironment for the development of CIN.

## Figures and Tables

**Table 1:** Sociodemographic and socioeconomic characteristics of participants.

Variables	Pap smear result, *n* (%)				*p* value
	NIL (*n* = 143)	CIN 1 (*n* = 12)	CIN 2 (*n* = 2)	Total (*n* = 157)	
Age groups					<0.001
21-30	39 (27.3)	0 (0.0)	0 (0.0)	39 (24.8)	
31-40	40 (28.0)	0 (0.0)	0 (0.0)	40 (25.5)	
41-50	31 (21.7)	4 (33.3)	0 (0.0)	35 (22.3)	
≥51	33 (23.1)	8 (66.7)	2 (100.0)	43 (27.4)	
Marital status					0.317
Single	27 (18.9)	0 (0.0)	0 (0.0)	27 (17.2)	
Married	70 (49.0)	9 (75.0)	2 (100.0)	81 (51.6)	
Cohabiting	17 (11.9)	3 (25.0)	0 (0.0)	20 (12.1)	
Divorced	10 (6.9)	0 (0.0)	0 (0.0)	10 (12.7)	
Widowed	19 (13.3)	0 (0.0)	0 (0.0)	19 (6.4)	
Educational level					0.783
Tertiary	11 (7.7)	0 (0.0)	0 (0.0)	11 (7.0)	
SHS	10 (7.0)	0 (0.0)	0 (0.0)	10 (6.4)	
JHS	73 (51.0)	6 (50.0)	2 (100.0)	81 (51.6)	
Primary	29 (20.3)	5 (41.7)	0 (0.0)	34 (21.7)	
No formal education	20 (14.0)	1 (8.3)	0 (0.0)	21 (13.4)	
Employment					0.891
Civil servant	8 (5.6)	0 (0.0)	0 (0.0)	8 (5.1)	
Self-employed	20 (14.0)	2 (16.6)	0 (0.0)	22 (14.0)	
Trading	59 (41.3)	5 (41.7)	2 (100.0)	66 (42.0)	
Farming/fishing	43 (30.1)	5 (41.7)	0 (0.0)	48 (30.6)	
No occupation	13 (9.1)	0 (0.0)	0 (0.0)	13 (8.3)	
Monthly income (Gh₵)					0.488
≤500	128 (89.5)	11 (91.7)	2 (100.0)	141 (89.8)	
600-1000	11 (7.7)	0 (0.0)	0 (0.0)	11 (7.0)	
1100-1500	2 (1.4)	1 (8.3)	0 (0.0)	3 (1.9)	
>1500	2 (1.4)	0 (0.0)	0 (0.0)	2 (1.3)	
Age at 1st sex (years)					0.417
≤16	31 (21.7)	4 (33.3)	0 (0.0)	35 (22.3)	
17-20	93 (65.0)	8 (66.7)	1 (50.0)	102 (65.0)	
21-24	16 (11.2)	0 (0.0)	1 (50.0)	17 (10.8)	
≥25	3 (2.1)	0 (0.0)	0 (0.0)	3 (1.9)	
Lifetime no. of sexual partners					0.909
1	20 (14.0)	2 (16.7)	0 (0.0)	22 (14.0)	
2-3	97 (67.8)	9 (75.0)	2 (100.0)	108 (68.8)	
≥4	26 (18.2)	1 (8.3)	0 (0.0)	27 (17.2)	
Alcohol consumption					0.38
Daily	6 (4.2)	0 (0.0)	0 (0.0)	6 (3.8)	
Occasional	36 (25.2)	1 (8.3)	1 (50.0)	38 (24.2)	
Never	101 (70.6)	11 (91.7)	1 (50.0)	113 (72.0)	
Hormonal contraception use					0.548
Ever^a^/current	75 (52.4)	7 (58.3)	2 (100.0)	84 (53.5)	
Never	68 (47.6)	5 (41.7)	0 (0.0)	73 (46.5)	
Number of full-term pregnancies					0.316
0	7 (4.9)	0 (0.0)	0 (0.0)	7 (4.5)	
1-3	80 (55.9)	5 (41.7)	0 (0.0)	85 (54.1)	
≥4	56 (39.2)	7 (58.3)	2 (100.0)	65 (41.4)	

The level of significance was established using chi-square and Fischer's exact tests. NIL = negative for intraepithelial lesions; CIN 1 = low-grade intraepithelial lesions; CIN 2 = high-grade intraepithelial lesions. ^a^Ever taken contraceptives within the previous 10 years.

**Table 2:** Bivariate analyses of CIN among participants associated with certain risk characteristics.

Variables	CIN (*N* = 14)	OR (95% CI)	*p* value
Age (years)			
≤46	12 (19.4)	1	
>46	2 (2.1)	11.16 (2.402-51.844)	0.002
Education			
Formal	13 (9.6)	1	
Nonformal	1 (4.8)	0.473 (0.586-3.818)	0.482
Income			
≥Gh₵ 1000.00	1(20.0)	1	
≤Gh₵ 1000.00	13 (8.6)	0.374 (0.039-3.599)	0.395
Age at 1st sex			
>20 years	1 (5.0)	1	
≤20 years	13 (9.5)	1.992 (0.246-16.112)	0.518
Lifetime number of partners			
1	2 (9.1)	1	
3-4	11 (10.2)	1.134 (0.233-5.515)	0.876
≥4	1 (3.7)	0.385 (0.033-4.548)	0.448
Contraceptive use			
Never used	5 (6.8)	1	
Ever/current use	9 (10.7)	1.632 (0.521-5.110)	0.400
No. of full-term pregnancies			
0-3	5 (5.4)	1	
≥4	9 (13.8)	2.796 (0.891-8.776)	0.078
Alcohol use			
Ever taken	2 (4.5)	1	
Never	12 (10.6)	2.495 (0.535-11.634)	0.244

**Table 3:** Distribution of participants based on CIN and vaginal infection.

Vaginal infection	Pap smear result, *n* (%)			*p* value
	Total (*n* = 157)	NIL (*n* = 143)	CIN 1+ (*n* = 14)	
No bacterial growth	24 (15.3)	24 (16.8)	0 (0.0)	0.130
Candida infection	49 (31.2)	40 (28.0)	9 (64.3)	0.012
Bacterial vaginosis	14 (8.9)	8 (5.6)	6 (42.9)	0.003
Other bacterial infection	101 (64.3)	91 (63.6)	10 (71.4)	0.772
*Lactobacillus* spp.	12 (11.9)	12 (13.2)	0 (0.0)	0.603
*Staphylococcus aureus*	22 (21.8)	16 (17.6)	6(60.0)	0.005
Group A *Streptococcus* spp.	4 (4.0)	4 (4.4)	0 (0.0)	>0.999
*Escherichia coli*	32 (31.7)	28 (30.8)	4 (40.0)	0.486
*Pseudomonas* sp.	11 (10.9)	11 (12.1)	0 (0.0)	0.596
*Citrobacter* spp.	16 (15.8)	12 (13.2)	4 (40.0)	0.017
*Morganella morganii*	8 (7.9)	4 (4.4)	4 (40.0)	0.002
*Klebsiella* spp.	5 (5.0)	4 (4.4)	1 (10.0)	0.377

The level of significance was established using Fischer's exact tests. *p* values reported are two-sided. NIL = negative for cervical intraepithelial neoplasia; CIN 1+ = low-grade squamous intraepithelial lesion (CIN 1) and high-grade squamous intraepithelial lesion (CIN 2).

**Table 4:** Mean concentrations of serum inflammatory cytokines.

Serum cytokines	Mean concentration (pg/ml)		
	NIL (*N* = 143)	CIN 1 (*N* = 12)	CIN 2 (*N* = 2)
IL-10	11.60 ± 3.519	13.61 ± 3.648 ns	15.11 ± 4.702 ns
IL-6 U	3.71 ± 2.018	3.50 ± 1.952 ns	2.06 ± 1.293 ns
IL-4	17.31 ± 6.518	19.82 ± 7.612 ns	16.27 ± 8.357 ns
IFN-*γ*	7.42 ± 2.556	8.55 ± 2.439 ns	6.62 ± 3.956 ns
TNF-*α*	38.95 ± 19.478	48.73 ± 18.77^∗∗^	49.03 ± 0.423 ns

Concentration values are *mean* ± *SD*. The level of significance was established using a two-way analysis of variance (ANOVA) followed by Bonferroni's multiple comparison test. ns implies *p* > 0.05 for no significant relationship between any of the column variables—cases (CIN 1 and/or CIN 2) and the control group (NIL)—within a row. ^∗∗^ represent *p* ≤ 0.001 implying a significant relationship between NIL and CIN 1 group for TNF-*α* concentration only. U = unit of ng/l.

**Table 5:** The concentration of cytokines among participants with CIN 1+ and vaginal infections.

Cytokines	Mean concentration (pg/ml)			
	NIL+NBG (*n* = 24)	RTI (*n* = 119)	CIN 1+RTI (*n* = 12)	CIN 2+RTI (*n* = 2)
IL-10	9.22 ± 3.91	9.98 ± 1.85 ns	13.61 ± 3.648 ns	15.11 ± 4.70 ns
IL-6 U	3.75 ± 0.95	3.85 ± 2.64 ns	3.50 ± 1.952 ns	2.06 ± 1.29 ns
IL-4	18.68 ± 2.11	17.11 ± 4.88 ns	19.82 ± 7.612 ns	16.27 ± 8.36 ns
IFN-*γ*	6.52 ± 0.93	6.26 ± 1.78 ns	8.55 ± 2.439 ns	6.62 ± 3.96 ns
TNF-*α*	54.32 ± 9.50	31.99 ± 19.29^∗∗∗∗^	48.73 ± 18.77^#^	49.03 ± 0.42

Concentration values are mean ± SD. The level of significance was established using two-way analysis of variance (ANOVA) followed by Bonferroni's multiple comparison test. ns implies *p* > 0.05 for no significant relationship between any of the column variables—cases (CIN 1 and/or CIN 2) and the control group (NIL)—within a row. ^∗^ and # represent *p* ≤ 0.05 implying significant relationship between groups. ^∗^ implies a significant association between NIL+NBG and RTI groups for TNF-*α* concentrations. # implies significant association between RTI and CIN 1+RTI groups for TNF-*α* concentrations. U = unit of ng/l.

## Data Availability

All data of this study are available from the corresponding author upon request.
